# Determinants of double product: a cross-sectional study of urban residents in Japan

**DOI:** 10.1265/ehpm.23-00002

**Published:** 2023-06-20

**Authors:** Natsuko Nakagoshi, Sachimi Kubo, Yoko Nishida, Kazuyo Kuwabara, Aya Hirata, Mizuki Sata, Aya Higashiyama, Yoshimi Kubota, Takumi Hirata, Yukako Tatsumi, Kuniko Kawamura, Junji Miyazaki, Naomi Miyamatsu, Daisuke Sugiyama, Yoshihiro Miyamoto, Tomonori Okamura

**Affiliations:** 1 Department of Preventive Medicine and Public Health, Keio University School of Medicine, Tokyo, Japan; 2 Faculty of Human Sciences, Tezukayama Gakuin University, Osaka, Japan; 3 Osaka Institute of Public Health, Osaka, Japan; 4 Department of Hygiene, Wakayama Medical University, Wakayama, Japan; 5 Department of Environmental and Preventive Medicine, School of Medicine, Hyogo Medical University, Hyogo, Japan; 6 Institute for Clinical and Translational Science, Nara Medical University, Nara, Japan; 7 Department of Hygiene and Public Health, Teikyo University School of Medicine, Tokyo, Japan; 8 Center for Cluster Development and Coordination, Foundation for Biomedical Research and Innovation at Kobe, Hyogo, Japan; 9 Department of Clinical Nursing, Shiga University of Medical Science, Shiga, Japan; 10 Faculty of Nursing and Medical Care, Keio University, Kanagawa, Japan; 11 Open Innovation Center, National Cerebral and Cardiovascular Center, Osaka, Japan

**Keywords:** Double product, Systolic blood pressure, Heart rate, Insulin resistance, Amount of exercise, γ-GTP

## Abstract

**Background:**

The current study aimed to investigate the determinants of high double product (DP) by evaluating the association between resting DP, which is calculated as systolic blood pressure (SBP) multiplied by heart rate (HR), and blood test results and lifestyle factors.

**Methods:**

This research included 973 participants in the baseline survey of the KOBE study, which included a cohort of urban residents. The possible DP determinants were identified by examining the association between lifestyle factors and laboratory findings and DP by analyzing covariance adjusted for sex and age. Logistic regression analysis was performed with high DP (SBP × HR ≥ 9145 mmHg beats/min or quintile according to sex) as outcome and DP determinants as independent variables.

**Results:**

Age, hematocrit, and gamma-glutamyl transferase (log) level were positively associated with a high DP in both men and women. In addition, a high DP was positively associated with Homeostatic Model Assessment for Insulin Resistance score in women alone. Meanwhile, the amount of exercise was negatively associated with a high DP in men alone.

**Conclusions:**

High DP values at rest were associated with insulin resistance, gamma-glutamyl transferase, and the amount of exercise in participants without underlying disease.

**Supplementary information:**

The online version contains supplementary material available at https://doi.org/10.1265/ehpm.23-00002.

## Graphical Abstract

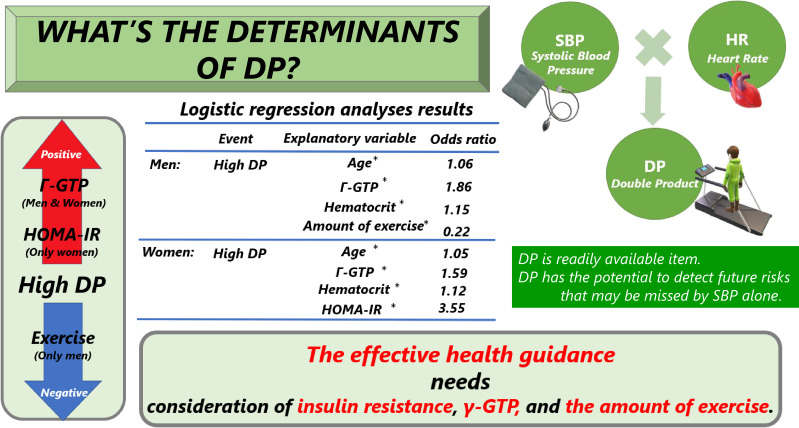

## Introduction

Double product (DP) is calculated by multiplying systolic blood pressure (SBP) and heart rate (HR) and is a surrogate marker of myocardial oxygen demand and cardiac work [1–3]. In clinical practice, DP is often used as an index of myocardial oxygen demand during exercise, and several studies have found that it useful in patients with heart disease [4–10]. However, in 2012, it was found that DP using home blood pressure, unlike that measured during exercise, is important clinically and is associated with CVD and all-cause mortality [11].

SBP and HR are risk factors of CVD [12–18]. And DP has been shown to be useful in subsequent heart failure exacerbation risk stratification [19] and is associated with all-cause, CVD- and stroke-related, and non-CVD mortality among Japanese [11]. However, there are only few reports have shown the determinants of DP in healthy individuals.

Since many cardiovascular diseases, including heart failure, have been found to be negatively correlated with their prognosis and heart rate [20], β-blockers with a slow beat effect have been widely used. However, their effectiveness on primary prevention for CVDs has not been different in preventing the development of CVDs compared to other antihypertensive drugs [21]. However, recently, the effectiveness of drugs for heart failure that lower only heart rate without affecting blood pressure has been recognized [22], so it is necessary to confirm the determinants of heart rate and DP again.

Therefore, this study examined the association between DP and lifestyle factors and blood test results in healthy individuals. We aimed to identify the determinants of high DP levels and provide useful insights for health guidance.

## Methods

### Study design and participants

We analyzed data collected from the baseline survey (2010/2011) of the Kobe Orthopedic and Biomedical Epidemiological (KOBE) study, an urban cohort research that used daily health as an index. The KOBE study aimed to identify the causes of daily health problems and to obtain lifestyle-related findings that can be used to maintain and prevent poor quality of life. A long-term observational study was conducted using these indicators as endpoints to elucidate factors associated with common diseases and worsening quality of life among the participants of the Kobe study. The baseline study was started in 2010, and follow-up surveys were conducted every 2 years. This study used data from a baseline survey conducted between July 2010 and December 2011. The details have been published in previous studies [23–27]. The brief description was as follows.

The eligibility criteria of the KOBE study were as follows: 1) participants aged 40–74 years during the start of the study, 2) those without a history of cancer or CVD, 3) those not receiving treatment for hypertension, diabetes, or dyslipidemia, and 4) those who were subjectively healthy. During the study, the participants were in a fasting state, and they underwent an interview, physical/physiological examination, and blood/urine tests. During the interview, the participants answered self-administered questionnaires about their lifestyle habits, including medication and medical history and smoking and drinking habits.

**Fig. 1:**
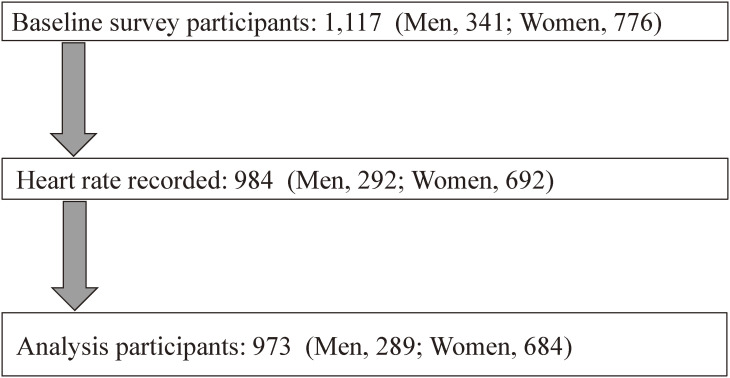
Study participants

Of 1117 participants who met the abovementioned criteria, 973 (289 men and 684 women) were included in the study. (Fig. 1).

### Assessment

Blood pressure and HR were evaluated twice using an automatic sphygmomanometer (BP-103i II; Nihon Colin) after a 5-min rest, and the average was obtained. DP was calculated as the product of the mean SBP and HR.

Blood samples were collected after 10 h of fasting, and all blood samples collected from the participants were evaluated at a laboratory (SRL Inc., https://www.srl-group.co.jp/english). Fasting blood glucose level (mg/dL) was assessed using the glucose oxidase method, and glycated hemoglobin (HbA1c) levels were evaluated using the latex agglutination test. High-density lipoprotein cholesterol (HDL-C), triglyceride, and creatinine levels were calculated using the enzymatic method, and low-density lipoprotein cholesterol (LDL-C) levels were evaluated using the Friedewald’s equation [28]. Hematocrit was determined by an automated analyzer indirectly. Aspartate aminotransferase (AST)/glutamic oxaloacetic transaminase (GOT) and alanine aminotransferase (ALT)/glutamic pyruvic transaminase (GPT) levels were calculated using the JSCC transferable method. The HOMA-IR score was calculated as follows [29].

$$\frac{\text{fasting blood glucose level }\left(\frac{\text{mg}}{\text{dL}}\right)\times \text{fasting insulin level }(\text{µU}/\text{mL})}{405}$$

Body mass index (BMI) was calculated by dividing body weight (kg) in light clothing by height in meters squared (m^2^). The estimated salt intake was equivalent to the estimated 24-h salt excretion as calculated by Tanaka et al. [30], who used spot urine, and corrected for creatinine content. Pure alcohol consumption per day was calculated from ethanol concentration and the amount of alcohol consumed from each alcoholic beverage. The average number of drinking times per week was determined [31]. Psychological stress was evaluated using the K6 depressive anxiety scale [32], and participants with scores of ≥ 5 were considered to have psychological stress.

The amount of exercise was calculated as metabolic equivalents (METs·hour/month) by investigating the type of exercise performed at least once a month, duration (min/day), and frequency (days/month) using a questionnaire. As described in a previous study [33], individual METs were calculated using “the Exercise Standards for Health Promotion 2006 New Exercise Standards/Exercise Guidelines” by the Japanese Ministry of Health, Labour and Welfare [34].

In this study, BMI; HOMA-IR score (log); hematocrit; fasting blood glucose, fasting insulin (log), HbA1c, gamma-glutamyl transpeptidase (γ-GTP, log), hemoglobin, triglyceride (log), LDL-C, HDL-C, AST (GOT, log), ALT (GPT, log), and creatinine levels; estimated salt intake; exercise habits; amount of exercise; daily walking time; drinking and smoking status; and K6 score were the candidate determinants of DP.

### Statistical analysis

Baseline characteristics were compared using the Student’s *t*-test and the χ2 test, and data were presented as mean (standard deviation) for continuous data and numerical values (percentages) for categorical data. To identify the candidate determinants of high DP, the association between laboratory findings and lifestyle factors and DP was evaluated by analyzing covariance adjusted for age and sex. Then, logistic regression analysis was performed using candidate factors as independent variables and high DP as a dependent variable. Based on a cohort study of Japanese community residents [11], high value was defined as a DP of ≥ 9145 mmHg × beats/min. Furthermore, additional analysis was performed based on the same study. A DP of 9145 mmHg × beats/min was the index calculated as the fifth quintile of home blood pressure. Thus, the fifth quintile of DP (men: ≥ 9181 mmHg × beats/min; women: ≥ 8805 mmHg × beats/min) was used as a dependent variable.

In the analysis of covariance, DP quartiles were used as a fixed factor. The association between DP and BMI, blood laboratory data (HOMA-IR score [log], hematocrit, and fasting blood glucose, fasting insulin [log], HbA1c, γ-GTP [log], hemoglobin, triglyceride hemoglobin, triglyceride [log], LDL-C, HDL-C, AST [GOT, log], ALT [GPT, log], and creatinine levels), estimated salt intake (g/day), exercise habits (non-exercise/regular exercise), amount of exercise, walking time (< 30 min, 30 min to < 1 h, 1 to < 2 h, and > 2 h), drinking status (nondrinking, previous drinking, current drinking ≤ 20 g net alcohol/day, and current drinking > 20 g net alcohol/day), smoking status (non-smoking, previous smoking, current smoking < 10 times/day, and current smoking ≥ 10 times/day), and K6 score was assessed. In the logistic regression analysis, the exercise group was divided into quartiles, and the non-exercise group was included in the first quartile.

The Hosmer & Lemeshow test was used to determine the goodness of fit. Analyses were performed using the Statistical Package for the Social Sciences software version 25 (IBM Inc., Tokyo, Japan), with a significance level of 5% on both sides.

## Results

### Characteristics of the participants

Table 1 shows the characteristics of 973 participants who were evaluated according to sex. The current study included 289 men and 684 women. All variables, except for HbA1c, ratio of regular exercise, and K6 score, significantly differed between men and women (all P < 0.05).

**Table 1:** Sex-specific baseline characteristics of the participants

**Characteristics**	**Men ** **(n = 289)**	**Women ** **(n = 684)**	**P values**
Age (years)	60.3 (9.1)	57.6 (8.9)	<0.001
BMI (kg/m^2^)	22.9 (2.7)	20.9 (2.6)	<0.001
HOMA-IR score^†^	0.7 (0.5–1.1)	0.7 (0.4–0.9)	<0.001
Fasting blood glucose level (mg/dL)	93.5 (13.6)	88.1 (12.7)	<0.001
Fasting insulin level (µIU/mL)^†^	3.3 (2.3–4.7)	3.0 (2.1–4.0)	0.003
HbA1c: NGSP (%)	5.6 (0.6)	5.6 (0.4)	0.302
γ-GTP level (U/L)^†^	32.0 (22.0–48.0)	18.0 (12.0–26.0)	<0.001
Hematocrit level (%)	43.9 (3.0)	39.5 (3.0)	<0.001
Hemoglobin level (g/dL)	14.8 (1.0)	13.2 (1.0)	<0.001
Triglyceride level (mg/dL)^†^	87.0 (64.0–120.5)	69.0 (53.0–96.8)	<0.001
LDL-C level (mg/dL)	123.6 (27.2)	133.9 (28.9)	<0.001
HDL-C level (mg/dL)	61.0 (14.2)	71.8 (15.5)	<0.001
AST (GOT) level (U/L)^†^	22.0 (19.0–25.0)	20.5 (18.0–24.0)	<0.001
ALT (GPT) level (U/L)^†^	20.0 (16.0–26.5)	17.0 (13.0–21.0)	<0.001
Creatinine level (mg/dL)	0.8 (0.01)	0.6 (0.01)	<0.001
Estimated salt intake (g/day)	8.9 (2.0)	8.2 (1.8)	<0.001
Regular exercise (%)	243 (84.1)	565 (82.6)	0.640
Amount of exercise (METs·h/month)^†^	72.5 (20.0–148.5)	40.6 (12.0–99.7)	<0.001
Walking time/day (%: ≥1 h)	161 (55.8)	452 (66.1%)	0.003
Current drinker (%)	220 (76.1)	256 (37.4)	<0.001
Current smoker (%)	36 (12.5)	14 (2.0)	<0.001
K6 score (point)^†^	1.0 (0.0–4.0)	2.0 (0.0–4.0)	0.295
DBP (mmHg)	70.5 (10.1)	68.8 (14.0)	<0.001
SBP (mmHg)	122.4 (17.1)	112.8 (16.6)	<0.001
HR (beat/min)	63.8 (9.5)	67.0 (9.2)	<0.001
DP (mmHg·HR)	7842.8 (1788.1)	7585.7 (1700.1)	0.034

† indicates that the HOMA-IR score, fasting insulin, γ-GTP, triglyceride, AST (GOT), ALT (GPT) levels, amount of exercise, and K6 score were presented as median (interquartile range). BMI: Body Mass Index, HOMA-IR: Homeostasis Model Assessment for Insulin Resistance, HbA1c: Hemoglobin A1c, γ-GTP: Gamma-Glutamyl Transpeptidase, LDL-C: Low-Density Lipoprotein Cholesterol, HDL-C: High-Density Lipoprotein Cholesterol, AST: Aspartate Aminotransferase, ALT: Alanine Aminotransferase, DBP: Diastolic Blood Pressure, SBP: Systolic Blood Pressure, HR: Heart Rate, DP: Double Product.

### Examination of factors associated with a high DP

Preliminary age-adjusted analysis of covariance showed that the HOMA-IR score (log) was significantly positively associated with DP in both men and women, and the amount of exercise was negatively associated with DP. Thus, these were extracted as candidate factors for determining high DP (Additional files 1–2). Further multivariate analyses were conducted, and factors that were significantly associated with DP were adjusted for each other. However, the significant positive association between DP and HOMA-IR score and the significant negative association between DP and the amount of exercise remained for both men and women (Additional file 3).

In addition, hematocrit and γ-GTP (log), hemoglobin, and triglyceride (log) levels were extracted as candidate factors for men based on the covariance analysis results (Additional file 1). In women, we added BMI, γ-GTP (log), hematocrit, hemoglobin, and triglyceride (log) levels, and estimated salt intake as candidate factors (Additional file 2). At this time, if there was no linear association between the dependent and independent variables, they were excluded as candidate factors even if significantly related. In the analysis of covariance, fasting insulin level (log) was also significantly positively associated in both men and women. However, it was excluded from the independent variables in the logistic regression analysis because of its strong association with HOMA-IR score. Moreover, there was a strong association between hematocrit and hemoglobin levels. Nevertheless, only hematocrit was selected and entered as an independent variable. Table 2 Model A shows the results of the logistic regression analysis.

**Table 2:** Logistic regression analysis results (high DP for men and women)

**Model A**	**Explanatory variable**	**Odds ratio**	**(95%CI)**		**Hosmer & Lemeshow test**	
	** Comparison/reference**		**Lower**	**Upper**		
Men: High DP (≥ 9145 mmHg × beats/min)						
	Age (years)*	1.06	1.02	1.10		
	HOMA-IR score (log)	1.37	0.76	2.48		
	γ-GTP level (IU/L) (log)*	1.86	1.16	2.99		
	Hematocrit (%)*	1.15	1.03	1.29		
	Triglyceride level (mg/dL) (log)	0.83	0.37	1.84		
	Amount of exercise (METs·h/month)					
	Medium (Q2)/low (Q1)·non	0.67	0.30	1.49		
	Medium (Q3)/low (Q1)·non	0.66	0.29	1.50		
	High (Q4)/low (Q1)·non*	0.22	0.08	0.64	4.83	(d.f. = 8) p = 0.78

Women: High DP (≥ 9145 mmHg × beats/min)						
	Age (years)*	1.05	1.02	1.08		
	BMI (kg/m^2^)	0.96	0.87	1.06		
	HOMA-IR score (log)*	3.55	2.03	6.22		
	γ-GTP level (IU/L) (log)*	1.59	1.08	2.35		
	Hematocrit (%)*	1.12	1.03	1.22		
	Triglyceride level (mg/dL) (log)	0.95	0.55	1.65		
	Estimated salt intake (g/day)	1,07	0.94	1.22		
	Amount of exercise (METs·h/month)					
	Medium (Q2)/low (Q1)·non	0.81	0.43	1.51		
	Medium (Q3)/low (Q1)·non	0.65	0.34	1.23		
	High (Q4)/low (Q1)·non	0.79	0.42	1.50	12.64	(d.f. = 8) p = 0.13

**Model B**	**Explanatory variable**	**Odds ratio**	**(95%CI)**		**Hosmer & Lemeshow test**	
	** Comparison/reference**		**Lower**	**Upper**		

Men: High DP = Q5 (≥ 9181 mmHg × beats/min)						
	Age (years)*	1.07	1.03	1.12		
	HOMA-IR score (log)	1.60	0.85	3.00		
	γ-GTP (IU/L) level (log)*	2.04	1.23	3.36		
	Hematocrit (%)*	1.18	1.05	1.33		
	Triglyceride level (mg/dL) (log)	0.51	0.21	1.21		
	Amount of exercise (METs·hour/month)					
	Medium (Q2)/low (Q1)·non	0.53	0.23	1.23		
	Medium (Q3)/low (Q1)·non	0.47	0.20	1.14		
	High (Q4)/low (Q1)·non*	0.17	0.05	0.51	6.63	(d.f. = 8) p = 0.58

Women: High DP = Q5 (≥ 8805 mmHg × beats/min)						
	Age (years)*	1.05	1.02	1.08		
	BMI (kg/m^2^)	0.97	0.88	1.05		
	HOMA-IR score (log)*	3.57	2.13	5.96		
	γ-GTP level (IU/L) (log)	1.33	0.92	1.92		
	Hematocrit (%)*	1.09	1.01	1.18		
	Triglyceride level (mg/dL) (log)	0.84	0.51	1.40		
	Estimated salt intake (g/day)	1.07	0.95	1.21		
	Amount of exercise (METs·h/month)					
	Medium (Q2)/low (Q1)·non	0.96	0.56	1.67		
	Medium (Q3)/low (Q1)·non	0.57	0.31	1.03		
	High (Q4)/low (Q1)·non	0.73	0.40	1.31	9.69	(d.f. = 8) p = 0.29

* indicates that there was a significant association between a high DP. BMI: Body Mass Index, HOMA-IR: Homeostasis Model Assessment for Insulin Resistance, γ-GTP: Gamma-Glutamyl Transpeptidase, DP: Double Product. Amount of exercise – non: non-exercise, low (Q1): < 45.1 (METs·h/month), Medium (Q2): 45.1–100.4 (METs·h/month), Medium (Q3): 100.5–160.6 (METs·h/month), High (Q4): ≥160.7 (METs·h/month) in men and non: non-exercise, low (Q1): < 28.6 (METs·h/month), Medium (Q2): 28.7–61.6 (METs·h/month), Medium (Q3): 61.7–120.0 (METs·h/month), High (Q4): ≥160.1 (METs·h/month) in women. DP – Q5: ≥ 9181 mmHg × beats/min in men and Q5: ≥ 8805 mmHg × beats/min in women.

In men, age (odds ratio [OR] = 1.06, 95% confidence interval [CI] = 1.02–1.10]), γ-GTP level (log) (OR = 1.86, 95% CI = 1.16–2.99), and hematocrit (OR = 1.15, 95% CI = 1.02–1.29) were positively associated with a high DP, and high levels of exercise (Q4) were negatively associated with a high DP (OR = 0.22 [95% CI = 0.08–0.64]). In women, age (OR = 1.05, 95% CI = 1.02–1.08), HOMA-IR (log) score (OR = 3.55, 95% CI = 2.03–6.22), γ-GTP level (log) (OR = 1.59, 95% CI = 1.08–2.35), and hematocrit (OR = 1.12, 95% CI = 1.03–1.22) were positively associated with a high DP. Furthermore, the logistic regression analysis results with quintile as outcome were similar as in Model A, except that the effect of γ-GTP (log) in women was eliminated (Table 2 Model B). Moreover, analysis was performed with hematocrit and hemoglobin level swapped, and the results were similar. In total, 65 men (22.5%) and 109 women (15.9%) corresponded to 9145 mmHg × beats/min or higher. The fifth quintile comprised 58 men (20.1%) and 136 women (19.9%).

## Discussion

Age, hematocrit, and γ-GTP (log) level were positively associated with a high DP in both men and women. HOMA-IR score (log) was positively associated with a high DP in women, and the amount of exercise was negatively associated with a high DP in men.

The findings of this study can be explained by previous studies. Hasegawa et al. conducted a cross-sectional study of 2736 Japanese men and women in their 40s and 60s living in central Tokyo. Results showed that sex, SBP, DBP, serum triglyceride and fasting blood glucose levels, and white blood cell count were significantly associated with HR [35]. Further, Shigetoh et al. reported that a high HR was associated with the development of obesity and diabetes for over 20 years [36]. However, these studies did not consider HOMA-IR score, an indicator of insulin resistance [37–38], which is associated with increased sympathetic nervous system activity [39]. Further, sympathetic nervous system activity is synergistically associated with the accumulation of metabolic syndrome risk factors [40]. Resting HR is a convenient index of sympathetic nervous system activity [36], and DP is a multiplier of SBP and HR. Hence, increased sympathetic activity may be associated with impaired glucose tolerance.

A significant positive association between hematocrit and a high DP has also been observed. The participants in this study did not include those with polycythemia vera, and their hematocrit value was within approximately normal limits. Stress polycythemia could have been present due to sympathetic tone [41], which can be a common cause of increased resting heart rate. The association between hematocrit and HR in hot and cold seasons was comparable. Therefore, it is unlikely that the effect of hematocrit reflects dehydration alone after fasting for >12 h.

Furthermore, in this study, a high DP was associated with γ-GTP levels in both men and women. γ-GTP level is correlated with drinking [42], which is a risk factor of hypertension [40]. Therefore, the result might have been influenced by drinking. However, the association between drinking status and DP is not significant in this study. Li et al. have revealed that γ-GTP is positively associated with CVD-related mortality, even in people who never drink, based on a longitudinal study that integrates multiple cohort studies in Japan [43]. Notably, γ-GTP may be an independent risk factor of overall CVD-related mortality regardless of drunkenness. γ-GTP can be associated with fatty liver and inflammation. Therefore, further validation of the association between γ-GTP levels and a high DP should be performed.

HOMA-IR score, similar to HR determinants, was mainly the determinant of DP proposed in our study, and factors in previous studies were extracted. Age and γ-GTP, which are the determinants of high DP values, similar to SBP determinants, were identified. DP is a product of SBP and HR, and the determinants could involve known factors from both. However, this study aimed to validate whether DP is similar to the determinant of SBP and HR, or which determinant is similar. A high DP includes cases of a normal SBP but a high HR. Currently, the predictive power of HR is not as strong as SBP. Hence, the use of DP in physical examination can detect the risk of SBP being overlooked because it is normal.

Further data found no significant association between exercise habits and DP. In addition, there was no significant association between walking time and DP in both men and women. Therefore, it is difficult to assess physical activity by walking time alone. Several reports have shown that aerobic exercise above certain levels decreases SBP and HR [44–48]. However, no studies have examined the association between exercise and DP. In addition, there was a significantly negative association between DP and the amount of exercise in both men and women. A high DP was negatively associated with the amount of exercise in men alone. In this study, the highest quartile of the amount of exercise (≥160.7 [METs] h/month; median: 224.0; quartile range: 183.1–297.8) was negatively associated with a high DP in men, and it was converted to about 7.5 exercise (METs × hour) per day. This is equivalent to playing tennis for approximately 10 min daily or running for about 7–8 min daily. Therefore, appropriate exercise may lower DP.

This study had several limitations. First, it was cross-sectional in nature. Thus, causal relationships cannot be evaluated. Initially, the association between each exposure coefficient and DP must be actively assessed. When interpreting the study results, it is always necessary to cautiously determine whether a causal relationship exists. Second, the participants were health conscious and had a high amount of regular exercise and low BMI. Hence, caution should be taken when examining the external validity of the study results. However, it is important to identify the determinants of DP among healthy participants in terms of early primary prevention in the community. In addition, the inclusion of a large number of women could have caused selection bias. Third, the KOBE study examined daily walking time and habitual physical activity only. Therefore, the overall amount of physical activity throughout daily life was not evaluated. In the future, it will be necessary to assess the total amount of physical activity in daily life in addition to exercise habits and to examine the association between DP and exercise intensity. Further, a longitudinal study should be performed to examine the association between DP and CVD outcomes.

Calculating DP is very simple and requires no additional cost. From a hygienic and public health point of view, DP is expected to become a simple valuable evaluation item for the health status of the general population. Based on this study, glucose intolerance, alcohol consumption and low physical activity were considered to be valuable health determinants when we were focusing on DP and/or heart rate. Furthermore, recent studies indicated that forest bathing attenuated sympathetic nerve activity, and decrease blood pressure and heart rate [49–50]; thus, an environmental improvement approach may be effective. Further research should be warranted.

## Conclusion

DP takes into account the predictive power of HR for CVD and mortality and has the potential to detect future risks that may be missed by SBP alone. In this study, in a healthy Japanese population, high DP was positively associated with age, hematocrit, and γ-GTP in both men and women. Furthermore, it was associated with higher HOMA-IR in women and a lower amount of exercise in men. This study suggests that DP may be improved by decreasing insulin resistance and γ-GTP levels, i.e., avoiding excessive alcohol drinking, and increasing the amount of exercise.

## Abbreviations

DP: Double Product
SBP: Systolic Blood Pressure
HR: Heart Rate
DBP: Diastolic Blood Pressure
BMI: Body Mass Index
CVD: cardiovascular disease
HOMA-IR: Homeostasis Model Assessment for Insulin Resistance
KOBE: Kobe Orthopedic and Biomedical Epidemiological
HbA1c: Hemoglobin A1c
γ-GTP: Gamma-Glutamyl Transpeptidase
LDL-C: Low-Density Lipoprotein Cholesterol
HDL-C: High-Density Lipoprotein Cholesterol
AST: Aspartate Aminotransferase
ALT: Alanine Aminotransferase
GPT: Glutamic Pyruvic Transaminase
GOT: Glutamic Oxaloacetic transaminase
SPSS: Statistical Package for The Social Sciences

## Supplementary information

**Additional file 1:** Covariance analyses results adjusted for age (DP for men).

**Additional file 2:** Covariance analyses results adjusted for age (DP for women).

**Additional file 3:** Covariance analyses results adjusted for age and candidate factors (DP for men and women).

## Declarations

### Availability of data and material

The most relevant data are within the paper. Raw data cannot be made publicly available, as study participants did not consent to have their information freely accessible.

Based on these consents, the Ethics Committee of the Institute of Biomedical Research and Innovation at the Kobe Biomedical Innovation Cluster (approval no. 10–20) and Keio University School of Medicine inhibit any public data sharing because data contain potentially identifying or sensitive disease information. Data accession requests may be sent to the administration of the Ethics Committees. The data will be shared after a review of the purpose and with permission from the ethics committees. Data requests can be made to the corresponding author.
