# Correction to: Determinants of double product: a cross-sectional study of urban residents in Japan

**DOI:** 10.1265/ehpm.23-00220

**Published:** 2023-12-05

**Authors:** Natsuko Nakagoshi, Sachimi Kubo, Yoko Nishida, Kazuyo Kuwabara, Aya Hirata, Mizuki Sata, Aya Higashiyama, Yoshimi Kubota, Takumi Hirata, Yukako Tatsumi, Kuniko Kawamura, Junji Miyazaki, Naomi Miyamatsu, Daisuke Sugiyama, Yoshihiro Miyamoto, Tomonori Okamura

**Affiliations:** 1 Department of Preventive Medicine and Public Health, Keio University School of Medicine, Tokyo, Japan; 2 Faculty of Human Sciences, Tezukayama Gakuin University, Osaka, Japan; 3 Osaka Institute of Public Health, Osaka, Japan; 4 Department of Hygiene, Wakayama Medical University, Wakayama, Japan; 5 Department of Environmental and Preventive Medicine, School of Medicine, Hyogo Medical University, Hyogo, Japan; 6 Institute for Clinical and Translational Science, Nara Medical University, Nara, Japan; 7 Department of Hygiene and Public Health, Teikyo University School of Medicine, Tokyo, Japan; 8 Center for Cluster Development and Coordination, Foundation for Biomedical Research and Innovation at Kobe, Hyogo, Japan; 9 Department of Clinical Nursing, Shiga University of Medical Science, Shiga, Japan; 10 Faculty of Nursing and Medical Care, Keio University, Kanagawa, Japan; 11 Open Innovation Center, National Cerebral and Cardiovascular Center, Osaka, Japan

**Correction to: *Environ Health Prev Med* 28, 37 (2023)**

**https://doi.org/10.1265/ehpm.23-00002**

After the publication of this article [1], it was discovered that there was a typo in one sentence of the discussion.

Therefore, we will correct it as follows.

On line 47 of the discussion, it says “This is equivalent to playing tennis for approximately 10 min daily or running for about 7–8 min daily.”

This is corrected to “This is equivalent to playing tennis for approximately 1 hour daily or running for about 40 min daily.”
